# Impact of climate change on animal health and welfare

**DOI:** 10.1093/af/vfy030

**Published:** 2018-11-10

**Authors:** Nicola Lacetera

**Affiliations:** Department of Agriculture and Forest Sciences, Viterbo, Italy

**Keywords:** health, immunity, metabolism, microorganisms, vectors

**Implications**

- Climate change is expected to exert an overwhelming negative effect on livestock health and welfare. Several studies suggest that the expected increase of air temperatures might reduce the risk of death and improve health and welfare of humans and livestock living in areas with very cold winters.
- The negative effects of climate change on animal health and welfare will be the consequence of combined changes of air temperature, precipitation, frequency, and magnitude of extreme weather events and may be both direct and indirect.
- The direct effects of climate change may be due primarily to increased temperatures and frequency and intensity of heat waves. Depending on its intensity and duration, heat stress may affect livestock health by causing metabolic disruptions, oxidative stress, and immune suppression causing infections and death.
- The indirect effects of climate change are primarily those linked to quantity and quality of feedstuffs and drinking water and survival and distribution of pathogens and/or their vectors.
- Development and application of methods linking climate data with disease occurrence should be implemented to prevent and/or manage climate-associated diseases.

## Introduction

Climate is one of many factors with the potential to alter disease states and is expected to exert an overwhelming negative effect on the health of humans and animals (Rabinowitz and Conti, 2013). In addition, several studies suggested that the increase of temperature might reduce mortality and/or improve health and welfare related aspects in humans and livestock living in geographic areas with cold winters (Ballester et al., 2011; Rose et al., 2015).

**Figure 1.**
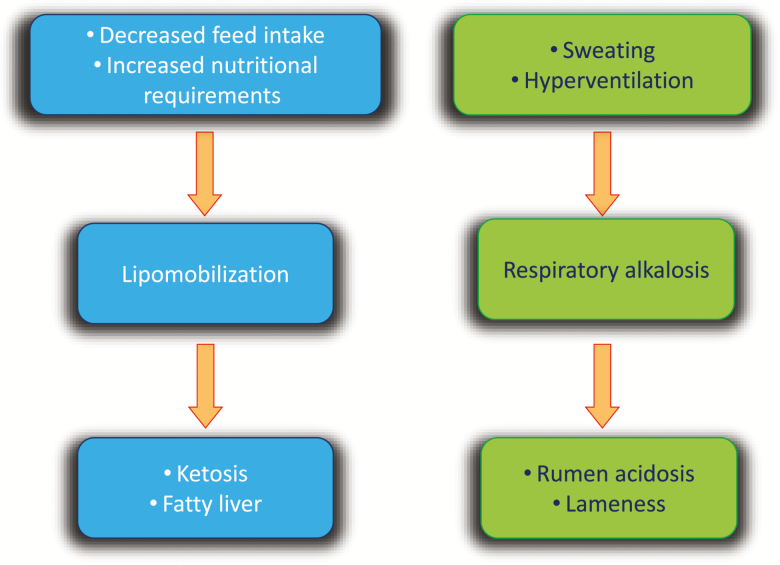
Schematic representation of the impact of climate change on animal health.

The effect of climate change on animal health may be either direct or indirect (Figure 1) and may be due primarily to changes in environmental conditions, which include air temperature, relative humidity, precipitation, and frequency and magnitude of extreme events (i.e., heat waves, severe droughts, extreme precipitation events, and coastal floods). Although this article focuses on the effects of environmental factors, it should be noted that factors leading to the effects of climate change on health are extremely complex, involving not only environmental forces, but also ecological and social aspects, economical interests, and individual and community behaviors (Forastiere, 2010).

The direct effects of climate change on health include temperature-related illness and death. Indirect impacts follow more intricate pathways and include those derived from the influence of climate on microbial density and distribution, distribution of vector-borne diseases, food and water shortages, or food-borne diseases (Lacetera et al., 2013). The aim of this article is to summarize the current state of knowledge regarding the influence of climate and climate change on the health of food-producing animals.

## Direct Effects

**Figure 2.**
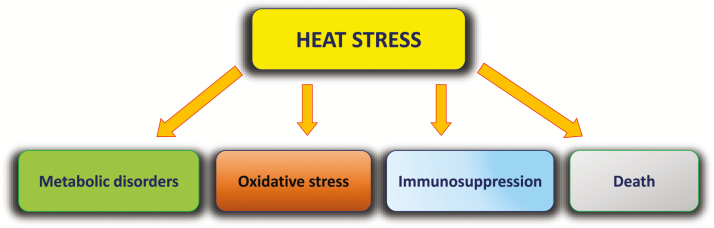
Schematic representation of the most frequent consequences of heat stress on animal health.

The direct effects of climate change on health may be due primarily to increased temperatures and frequency and intensity of heat waves (Gaughan et al., 2009). These effects are mediated by induction of heat stress conditions. Depending on its intensity and duration, heat stress may negatively affect livestock health by causing metabolic alterations, oxidative stress, immune suppression, and death (Figure 2).

## Metabolic Disorders

**Figure 3.**
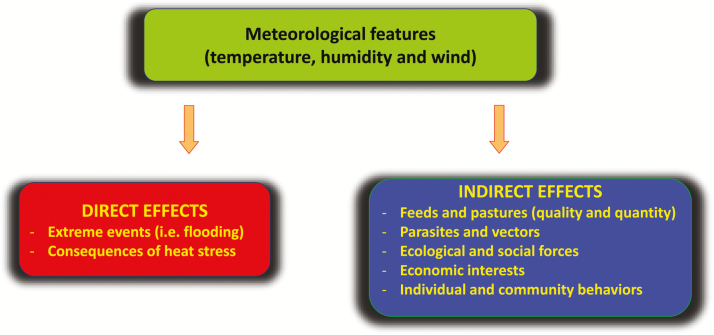
Schematic representation of some mechanisms through which heat stress may cause metabolic disorders in farm animals.

Homeothermic animals respond to high temperatures by increasing heat loss and reducing heat production in their attempt to avoid increased body temperature (hyperthermia). Such responses include an increase in respiratory and sweating rates and a decrease in feed intake. These physiological events may provide a significant contribution to explain the occurrence of metabolic disorders in heat-stressed animals (Figure 3).

Heat stress can contribute to the occurrence of lameness in dairy and beef cows (Shearer, 1999). Lameness in cattle may be defined as any foot abnormality that causes an animal to change the way that it walks. Lameness can be caused by a range of foot and leg conditions, themselves caused by disease, management, or environmental factors and is one of the most significant health, welfare, and productivity issues. The contribution of heat stress to lameness is perhaps due to ruminal acidosis or increased output of bicarbonate (Cook and Nordlund, 2009). Heat-stressed cattle eat less frequently during cooler times of the day, but they eat more at each feeding. Reduced feed intake during the hotter part of the day, followed by increased feeding when the ambient temperature cools down, can cause acidosis which is considered a major cause of laminitis (Shearer, 1999). As ambient temperatures rise, the respiratory rate increases with panting progressing to open-mouth breathing. A consequence is respiratory alkalosis resulting from a rapid loss of carbon dioxide. Cattle compensate by increasing urinary output of bicarbonate. Rumen buffering is affected by a decreased salivary bicarbonate pool. Lameness, with sole ulcers and white line disease, will appear in a few weeks to a few months after heat stress.

The reduction of feed intake combined with increased energy expenditure for maintenance may alter energy balance and explain why heat-stressed animals lose body weight and/or mobilize adipose tissue during heat stress. In particular, during summer, early lactating dairy cows are more likely to experience subclinical or clinical ketosis (Lacetera et al., 1996) and are at higher risk to develop liver lipidosis (Basiricò et al., 2009). Ketosis is a metabolic disease that occurs when the animal is in a severe state of negative energy balance, undergoes intense lipomobilization, and accumulates ketone bodies, which derive from incomplete catabolism of fat. Liver lipidosis is another consequence of the intense mobilization of fat from adipose tissue. Compromised liver function in heat-stressed cattle is testified by reduced albumin secretion and liver enzyme activities (Ronchi et al., 1999).

## Oxidative Stress

**Figure 4.**
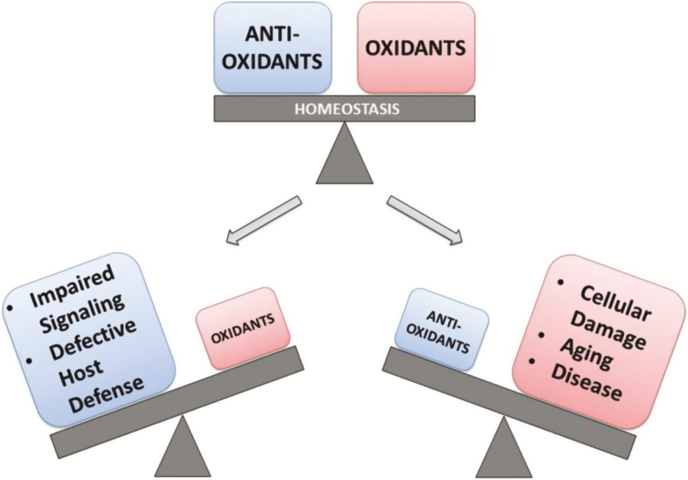
Balance between oxidants and antioxidants molecules in animal health and disease (from Knoefler et al., 2014).

In farm animals, oxidative stress may be involved in several pathological conditions, including conditions that are relevant for animal production and the general welfare of individuals (Lykkesfeldt and Svendsen, 2007). Oxidative stress results from an imbalance between oxidant and antioxidant molecules and may depend on the excess of oxidant and/or lack of antioxidant substances (Figure 4). In the last 10 to 15 yr, the involvement of heat stress in inducing oxidative stress in farm animals has received increasing interest (Bernabucci et al., 2002; Akbarian et al., 2016). The total antioxidant status concentrations in serum of heifers were lower in the summer than in the winter in peri- and postpartum periods (Mirzad et al., 2018). In mid-lactating cows, plasma values of reactive oxygen metabolite substances were increased during summer. Total carotenes and vitamin E were decreased during summer. Increased oxidant and decreased antioxidant molecules in blood during the hot summer season have been reported both in dairy and buffalo cows. Finally, heat stress has been associated with an increase of antioxidant enzyme activities (e.g., superoxide dismutase, catalase, and glutathione peroxidase), which has been interpreted as an adaptation response to increased levels of reactive oxygen species.

## Immune Suppression

The immune system has evolved as a complex of mechanisms to protect the host from invasion by pathogenic organisms. A number of factors may affect the proper functioning of the immune system (Lacetera, 2012). Several studies reported that heat stress may impair the function of the immune system in food-producing animals. Effects of heat stress on immune function are not always straightforward and may depend on the species, breed, genotype, age, social status, acclimation level, and intensity and duration of the exposure to the unfavorable conditions.

Immune suppression facilitates the occurrence of infections, which impairs reproductive efficiency, overall production efficiency, and may compromise animal welfare and increase the use of antimicrobials. Increased use of antimicrobials may lead to development of antimicrobial resistance in microorganisms.

Briefly, Regnier and Kelley (1981) reported that chronic exposure to heat stress impaired immune response in avian species. Nardone et al. (1997) indicated that severe heat stress reduced colostral immunoglobulins (IgG and IgA) in dairy cows with negative consequences on immunization and survival of newborn calves. Lacetera et al. (2005) described a dramatic depression in lymphocyte function in severely heat stressed peri-parturient dairy cows, which may increase their vulnerability to pathogens and also reduce the efficacy of vaccinations. Finally, Lecchi et al. (2016) reported that high temperatures impaired significantly the functionality of neutrophils, which have a central role in the protection of the mammary gland against infections. Mastitis is a major endemic disease of dairy cattle and usually occurs as an immune response to bacterial invasion of the teat canal or as a result of chemical, mechanical, or thermal injury to the cow’s udder. Several studies reported the increased occurrence of mastitis during the summer months (Morse et al., 1988; Waage et al., 1998). Results of a recent 2-yr study on the largest Italian dairy farm demonstrated that the greater risk of the occurrence of clinical mastitis in primiparous dairy cows was recorded in July (Vitali et al., 2016). Heat stress may improve the survival capability or growth of pathogens or their vectors (Chirico et al., 1997), and they may surely be involved in these important epidemiological findings. Further epidemiological studies are necessary to determine whether high environmental temperatures are associated with a higher incidence of other infections. The potential for impairment of immune cell function under hot environment supports the use of management practices (i.e., cooling, altered nutritional programs, improved animal hygiene, etc.), which may help to limit the increase of body temperature to prevent outbreaks of infections.

## Death

A series of studies have described a greater risk of mortality during the hottest months (Dechow and Goodling, 2008; Vitali et al., 2009) and an increased death rate during extreme weather events (Hahn et al., 2002; Vitali et al., 2015). High temperatures may cause heat stroke, heat exhaustion, heat syncope, heat cramps, and ultimately organ dysfunction. These heat-induced complications occur when the body temperature rises 3 to 4 °C above normal.

In an Indian study, Purusothaman et al. (2008) reported an increase of mortality in Mecheri sheep during summer season. Another series of studies on the effects of temperatures on mortality in farm animals described an increase of deaths during extreme weather events. Hahn and Mader (1997) and Hahn et al. (2002) described the impact on livestock from a weeklong heat wave in the mid-central United States during July 1995. A heat wave is generally defined as a prolonged period of excessively hot weather. It was also reported that during the severe and prolonged heat waves which occurred in Europe during summer 2003, over 35,000 people and thousands of pigs, poultry, and rabbits died in the French regions of Brittany and Pays-de-la-Loire (http://lists.envirolink.org/pipermail/ar-news/Week-of-Mon-20030804/004707.html). Vitali et al. (2015) indicated that summer mortality in dairy cows was greater during days in a heat wave compared with days not in a heat wave. Furthermore, the risk of mortality continued to be higher during the three days after the end of the heat wave. Mortality also increased with the length of the heat wave. Considering deaths stratified by age, cows up to 28 mo old were not affected by heat waves, whereas all the other age categories of cows (29 to 60, 61 to 96, and >96 mo) showed a greater mortality when exposed to a heat wave. The risk of death during a heat wave was higher in the early summer months. In particular, the highest risk of mortality was observed during a heat wave in June.

The temperature–humidity index combines temperature and humidity into a single value and is widely considered a useful tool to predict the effects of the environment on farm animals.

An epidemiological study with dairy cows (Vitali et al., 2009) indicated that 80 and 70 are the daily maximum and minimum temperature–humidity index values, respectively, above which heat-induced death rate increases. In addition, the same study indicated that 87 and 77 are the daily upper critical maximum and minimum temperature–humidity index, respectively, above which the risk of heat-induced death becomes maximum.

A recent study with swine in Italy reported the effects of month, length of the journey, and temperature–humidity index on mortality of heavy slaughter pigs (approximately 160 kg live weight) during transport and lairage (Vitali et al., 2014). The aggregated data of the summer vs. nonsummer months showed a greater risk of pigs dying during the hot season when considering both transport and lairage. The month with the greatest frequency of deaths was July, whereas the lower mortality risk ratios were recorded for January and March. The mortality risk ratio during transport increased significantly for journeys longer than 2 h. Finally, 78.5 and 73.6 temperature–humidity index were the thresholds above which the mortality rate increased significantly during transport and at lairage, respectively. In a long-term study on scenarios of temperature-related mortality in Europe, Ballester et al. (2011) predicted a change in the seasonality of mortality, with maximum monthly incidence progressively shifting from winter to summer from 1950 to 2100.

## Indirect Effects

As already described earlier, weather and climate change are likely to affect the biology and distribution of vector-borne infections. For example, temperature changes, global wind and precipitation patterns, and changes in relative humidity in temperate climates will affect positively the reproduction of insects and, consequently, their population density. Thus, some tropical diseases, especially those transmitted by insects, may probably move from their natural basin of endemic to other countries.

Simulating an increase of temperature values by 2 °C, a model tested by Wittmann et al. (2001) indicated the possibility of an extensive spread of *Culicoides imicola*, which represents the major vector of the bluetongue virus. This virus is responsible for an infectious arthropod-borne disease primarily of domestic and wild ruminants. Infection with bluetongue virus is common in a broad band across the world. Since 1990, this virus has spread considerably due to changing climatic and environmental conditions necessary to support the *Culicoides* vectors.

Another mechanism through which climate change may alter livestock and human health is represented by the favorable effects that high temperatures and moisture may exert on growth of mycotoxin-producing fungi. Growth of these fungi and the associated toxin production are closely related to the temperature and degree of moisture, which are dependent on weather conditions at harvest and techniques for drying and storage of grains (Frank, 1991). Mycotoxins can cause acute disease episodes when animals consume critical quantities of contaminated feeds. These mycotoxins may have a negative effect on specific tissues and organs such as liver, kidney, oral and gastric mucosa, brain, or reproductive tract. Most frequently, however, concentrations of mycotoxin in feeds are below those that can cause acute disease. At low concentrations, mycotoxins may reduce the growth rate of young animals. Some mycotoxins may interfere with the native mechanisms of disease resistance and may impair immunologic responsiveness, making the animals more susceptible to infection (Bernabucci et al., 2011).

Finally, other examples of how climate change may affect animal health are provided from parasitic diseases. In this context, gastrointestinal nematodes are important parasites of livestock, causing mortality and morbidity. Because a significant part of the life cycle of these parasites is completed outside of the host, their survival and development are susceptible to climate change. In this regard, a recent simulation study (Rose et al., 2015) predicted that future climatic data for a temperate region will have an opposite effect on annual infection pressure (increase or decrease) depending on the species of parasites.

## Conclusions

Although further epidemiological studies are needed, a significant amount of research has already demonstrated that climate change will affect animal health and welfare. Heat stress conditions as a result of global warming, high air temperatures, and higher frequency of extreme weather events and droughts may negatively affect animal health and welfare. Such effects may take place by direct and/or indirect mechanisms. Tools and techniques for an animal disease surveillance system to incorporate animal data with relevant climate conditions are also needed. Development and application of methodology to link climate data with disease surveillance systems should be implemented to improve prevention of diseases as well as mitigation and adaptation responses of animals to heat stress.

## About the Author

Nicola Lacetera graduated in Veterinary Medicine and earned a PhD in Veterinary Immunology from the University of Perugia, Italy. He joined the University of Tuscia (Viterbo, Italy) as an assistant professor in the Institute of Animal Science and currently is professor and head of Department of Agriculture and Forest Sciences. He was visiting professor at Caldwell Animal Hospital (USA, 1986), Colorado State University (USA, 1988 to 1989), University of Bristol (UK, 1994 and 1995), the Ecole Vétérinaire of Lion (France, 1997), and Commonwealth Scientific and Industrial Research Organization in Australia (2015). His research focuses on the interactions between metabolism, immunity, and health in dairy cattle and the effects of heat stress on health, performance, and risk of death in dairy cows.
