# Correction to: Impact of climate change on animal health and welfare

**DOI:** 10.1093/af/vfae002

**Published:** 2024-02-14

This is a correction to: Nicola Lacetera, Impact of climate change on animal health and welfare, *Animal Frontiers*, Volume 9, Issue 1, January 2019, Pages 26–31, https://doi.org/10.1093/af/vfy030

In the originally published online version of this manuscript, in the section **About The Author**, the first sentence should read: “Nicola Lacetera graduated in Veterinary Medicine from the University of Perugia, Italy.” instead of: “Nicola Lacetera graduated in Veterinary Medicine and earned a PhD in Veterinary Immunology from the University of Perugia, Italy.”

This emendation has been outlined only in this correction notice to preserve the version of record.

